# When Risk Persists: Two-Time Longitudinal Assessment of Healthcare Workers’ Exposure Risk in the Context of COVID-19

**DOI:** 10.3390/healthcare14030384

**Published:** 2026-02-03

**Authors:** Garyfallia Akrivouli, Dimitrios Papagiannis, Zoe Daniil, Ioannis C. Lampropoulos, Erasmia Rouka, Michael Spanos, Konstantinos I. Gourgoulianis, Foteini Malli

**Affiliations:** 1 Department of Nursing, University of Thessaly, Gaiopolis, 41500 Larissa, Greecei.ch.lampropoulos@gmail.com (I.C.L.); errouka@uth.gr (E.R.);; 2 Respiratory Medicine Department, University of Thessaly, Biopolis, 41110 Larissa, Greece

**Keywords:** COVID-19, healthcare workers, occupational exposure, infection prevention and control, risk assessment, personal protective equipment, prospective study, aerosol-generating procedures

## Abstract

**Highlights:**

**What are the main findings?**

- Classification of high-risk occupational exposure increased over time, potentially reflecting shifts in self-reported adherence to IPC practices.
- Adherence to key infection prevention and control practices declined, with nurses showing consistently higher risk.

**What are the implications of the main findings?**

- Occupational risk requires ongoing monitoring rather than single-time assessment.
- Findings suggest a need for ongoing attention to protective measures, as adherence declines over time.

**Abstract:**

**Background**: Healthcare workers (HCWs) have experienced sustained occupational exposure to SARS-CoV-2 throughout the COVID-19 pandemic. While infection prevention and control (IPC) practices have been widely implemented, limited prospective data exist on how occupational exposure risk and adherence to protective practices evolve over time, particularly beyond the acute phases of the pandemic. This study aimed to prospectively assess occupational and community exposure risk to COVID-19 among HCWs and to evaluate temporal changes in adherence to IPC practices during routine care and aerosol-generating procedures. **Methods**: A prospective observational study was conducted among HCWs from four public hospitals in the region of Thessaly, Greece. Eligible participants were HCWs who reported contact with suspected or confirmed COVID-19 cases. The data were collected at baseline (January–March 2022) and at a six-month follow-up using the World Health Organization’s “Risk Assessment and Management of Exposure of Health Care Workers in the Context of COVID-19” questionnaire. The instrument captured demographic characteristics, professional roles, occupational and community exposure, and adherence to IPC practices. **Results**: A total of 203 HCWs participated in the study. The overall proportion of HCWs reporting occupational exposure was 72.9% in both assessments. Among HCWs with occupational exposure (n = 148), the proportion classified as high-risk showed a statistically significant increase from 76% (95% CI: 0.6951–0.8320) at baseline to 88% (95% CI: 0.8258–0.9310) at follow-up (*p* = 0.010). This shift reflects a substantial effect size, with the odds of high-risk classification being more than double at follow-up (OR = 2.24). Nurses consistently demonstrated higher risk compared with physicians. The overall use of personal protective equipment remained high. However, adherence to several IPC practices declined over time, including removal and replacement of PPE according to protocol and frequent decontamination of high-touch surfaces. **Conclusions**: Occupational exposure risk among HCWs remained substantial and increased over time despite stable occupational exposure estimates. The observed decline in adherence to key IPC measures highlights the need for continuous monitoring and reinforcement of protective practices in healthcare settings.

## 1. Introduction

Healthcare workers (HCWs) have been disproportionately affected by the COVID-19 pandemic due to their sustained exposure to SARS-CoV-2 in clinical settings. From the early stages of the pandemic, HCWs faced elevated risks of infection, psychological burden, and occupational strain, particularly those involved in direct patient care and aerosol-generating procedures (AGPs) [1,2]. Despite the implementation of infection prevention and control (IPC) practices and vaccination, occupational exposure remains a critical concern, especially as adherence to protective measures may worsen over time.

Multiple studies have demonstrated that HCWs are at a higher risk of SARS-CoV-2 infection compared with the general population, with reported infection rates varying widely across settings and professional roles [3,4]. Nurses, in particular, have consistently shown higher infection risk, likely reflecting prolonged patient contact, frequent involvement in high-risk procedures, and workload intensity [5]. Age, sex, and type of HCW have been identified as potential modifiers of occupational risk [6]. Evidence suggests that aerosol-generating procedures are associated with a significantly higher exposure risk, particularly when adherence to personal protective equipment (PPE) protocols and hand hygiene practices are suboptimal [7]. The existing literature suggests that the adherence of HCWs to IPC practices during the COVID-19 pandemic is associated with reduced rates of SARS-CoV-2 seropositivity [8]. Real-world compliance with IPC measures during both routine care and AGPs has shown substantial variability, even in high-resource healthcare systems [9].

To support standardized evaluation of the exposure risk, the World Health Organization (WHO) developed the “Risk Assessment and Management of Exposure of Health Care Workers in the Context of COVID-19” tool, which has been widely adopted in epidemiological and occupational health research [10,11,12]. Studies applying this instrument have demonstrated its utility in identifying high-risk exposures, professional disparities, and gaps in adherence to IPC practices [12]. However, most available data are cross-sectional, limiting insight into temporal changes in risk patterns and behavioral compliance.

Prospective, as well as longitudinal, assessments of occupational exposure and adherence to IPC measures among HCWs remain scarce. The existing data indicate a decline in compliance with hand hygiene measures as the pandemic subsided [13]. Understanding how risk profiles evolve over time is essential for informing targeted interventions and ensuring continued adherence to IPC practices in the context of ongoing and future infectious disease threats. Although the intensity of the COVID-19 pandemic has diminished, adherence to IPC measures in healthcare settings remains a critical concern. Changes in risk perception over time may affect compliance, highlighting the importance of prospective evaluations of occupational exposure among HCWs. In the present study, we aimed to prospectively assess occupational and community exposure to COVID-19 among HCWs in public hospitals in Greece and evaluate changes in infection risk over a six-month period. Additionally, we sought to examine adherence to IPC measures during routine care and aerosol-generating procedures using a standardized WHO-based risk assessment framework.

## 2. Materials and Methods

### 2.1. Study Design and Population

The study involved healthcare workers from four public hospitals in the region of Thessaly, including one tertiary care facility and three secondary-level hospitals. The convenience sampling method was implemented. We included HCWs who had contact with individuals suspected of having COVID-19. HCWs that did not report contact with suspected or confirmed COVID-19 cases and those that did not provide consent to participate in the study were excluded. Ethical approval was obtained by the Institutional Review Board of the Nursing Department of the University of Thessaly (protocol code 449/08-04-2021). Participation was voluntary while all individuals gave written informed consent before inclusion in the study. The data collection occurred during routine staff meetings or shifts and anonymity was ensured. All procedures adhered to the ethical principles outlined in the Declaration of Helsinki for studies involving human participants. The design and reporting of the study follow the Strengthening the Reporting of Observational Studies in Epidemiology (STROBE) guidelines.

### 2.2. Measurements

We used the World Health Organization’s 2020 instrument titled “Risk assessment and management of exposure of healthcare workers in the context of COVID-19” as the primary instrument for evaluating COVID-19 exposure risk among healthcare personnel [10]. This tool is considered to be one of the most commonly utilized approaches for assessing COVID-19 risk in clinical environments. For the purposes of our study, the questionnaire was translated into Greek. A preliminary version of the questionnaire was pilot-tested in order to evaluate its clarity and feasibility. The questionnaire underwent a forward–backward translation process: it was initially translated from English into Greek, followed by a back-translation into English by an independent translator unfamiliar with the original. Differences between the versions were carefully reviewed and resolved to maintain accuracy and consistency with the source content. A pilot test was then conducted with 15 healthcare workers to evaluate the tool’s clarity and contextual relevance. The feedback from this group informed minor revisions to the questionnaire, and their responses were not included in the main data analysis. Completing the final instrument required approximately 10–20 min. Participants completed the questionnaire included in the appendix at two prospective time points: initially between January and March 2022 and again six months later.

As previously described in ref. [12], the questionnaire includes eight items on healthcare worker demographics, six items regarding interactions with patients with COVID-19, and five items on clinical activities performed with these patients within the healthcare setting. It also assesses adherence to IPC practices during patient care through seven items, as well as compliance with IPC measures during aerosol-generating procedures (i.e., tracheal intubation, nebulization, open airway suctioning, sputum collection, tracheotomy, bronchoscopy, and cardiopulmonary resuscitation) through an additional six items. Finally, two items recorded incidents involving exposure to biological materials.

In accordance with the design of the questionnaire [11], subjects were classified as having community exposure if they answered “yes” to any of the following: residing in the same household or sharing a classroom environment with an individual confirmed to have COVID-19 or traveling in close proximity (within 1 m) to a confirmed case in any form of transportation.

In the same context, occupational exposure was defined as previously described [11]. In more detail, occupational exposure to the COVID-19 virus was determined based on affirmative responses to any of the following activities involving patients with COVID-19: delivering direct care, being present during or performing aerosol-generating procedures, or having direct contact with the care environment of a confirmed COVID-19 patient. HCWs with occupational exposure to SARS-CoV-2 were classified into “high risk for COVID-19 virus infection” and “low risk of COVID-19 virus infection”, based on their reported adherence to IPC measures. Briefly, HCWs were considered at high risk if they failed to report consistent compliance (i.e., specifically, responding “always, as recommended”) in relation to key IPC practices while interacting with confirmed COVID-19 patients. These practices included the appropriate use of personal protective equipment (PPE) such as single-use gloves, medical masks, eye protection (goggles or face shields), and disposable gowns; proper donning and doffing of PPE; and performing hand hygiene at all critical moments (e.g., before and after patient contact, before cleaning procedures, after exposure to bodily fluids, and after touching patient surroundings) [11]. Regular decontamination (at least 3 times) of high-touch surfaces (at least three times daily) was also considered.

In cases involving aerosol-generating procedures (AGPs), HCWs were considered high-risk if they failed to respond “always, as recommended” to the recommended use of single-use gloves, N95 (or equivalent operator) masks, face shields or goggles/protective glasses, disposable gowns, waterproof aprons, PPE handling according to protocol, hand hygiene (before and after touching a COVID-19 patient), and regular (at least 3 times per day) high-touch surface decontamination. Additionally, any reported incident of exposure to respiratory secretions or bodily fluids from a COVID-19 patient was also considered high-risk. All other responses resulted in a low-risk classification.

### 2.3. Statistical Analysis

All the necessary analyses were performed using the Statistical Package IBM SPSS Statistics 29.0 (Chicago, IL, USA). Descriptive statistics were used to describe the baseline characteristics of HCWs. For data with normal and non-normal distribution, the means and standard deviations, as well as the medians and interquartile ranges, were calculated, respectively. The McNemar test was performed to determine the dependence between the categorical variables. For any longitudinal comparisons involving continuous data, we have applied the paired-samples *t*-test. The significance level was set at 0.05 for each of the above tests. All statistical analyses were performed using the complete-case analysis (listwise deletion). This is the standard procedure in SPSS, where participants with missing values in the variables of interest are automatically excluded from the specific calculations. Therefore, the reported associations and *p*-values were based on the available complete data for each variable.

## 3. Results

### 3.1. Occupational Risk Results

We recruited 203 HCWs from four hospitals (37.91% nurses, 27.47% physicians, and 10.99% nursing assistants) with a mean age of 45.84 ± 7.64 years (Table 1). All 203 participants completed both the baseline and the follow-up assessment. The majority (69.5%) of the HCWs were women. At the first assessment, 38.91% of the participants reported a history of a previous SARS-CoV-2 infection, while 6.3% of them needed hospitalization. Occupational exposure was documented in 72.90% of participants at both time assessments. Among HCWs with occupational exposure (n = 148), the proportion classified as high-risk showed a statistically significant increase from 76% (95% CI: 0.6951–0.8320) at baseline to 88% (95% CI: 0.8258–0.9310) at follow-up (*p* = 0.010). This shift reflects a substantial effect size, with the odds of high-risk classification being more than double at follow-up (OR = 2.24). Community exposure was reported in 55% at baseline and in 54% during the follow-up, 6 months later.

Women appeared to have higher occupational exposure to COVID-19 at both time points, but the difference was not statistically significant. At baseline, high-risk exposure was observed in 59.6% of males (n = 28/47) and 55.3% of females (n = 78/141) (*p* = 0.166) (Table 2). At the 6-month follow-up, the within-group risk rates remained comparable between genders, with high-risk exposure affecting 61.7% of males (n = 29/47) and 64.5% of females (n = 91/141) (*p* = 0.60). These results indicated that, while women represented the numerical majority of the high-risk group, the actual probability of being classified as high-risk did not differ significantly by gender.

At baseline, high-risk exposure was observed in 59.6% of males and 55.3% of females (*p* = 0.484) (Table 2). Six months later, HR exposure remained similar, affecting 23.6% of males and 74.0% of females (*p* = 0.604). Seventeen HCWs changed from high-risk to low and 27 from low to high-risk, as shown in Table 3 and Table 4.

At baseline, most participants were aged 35 years or older (79.3%), and this age group accounted for a higher proportion of occupational exposure (86.8%) compared with those aged ≥35 years (13.2%) (*p* = 0.030). In contrast, no significant association was observed between age and risk of COVID-19 infection at baseline, with similar distributions of younger and older participants across low-risk (85.2% vs. 14.8%) and high-risk categories (87.3% vs. 12.7%; *p* = 0.777). At the 6-month assessment, age was no longer significantly associated with either occupational exposure (*p* = 0.160) or risk of COVID-19 infection (*p* = 0.428). Participants younger than 35 continued to represent the majority of both exposed (86.8%) and high-risk individuals (86.0%), while those aged ≥35 accounted for a smaller proportion across exposure and risk categories.

The risk of COVID-19 infection differed significantly by healthcare worker category at both time points (*p* < 0.001). At baseline, nurses exhibited a higher proportion of high-risk (HR) classification compared with doctors (84.0% vs. 83.6%). This pattern persisted at the 6-month follow-up, with the nurses continuing to show a higher HR proportion than doctors (80.2% vs. 87.3%) (Table 2).

### 3.2. Adherence to IPC Measures

#### 3.2.1. Adherence to IPC Measures During Healthcare Interactions

During healthcare interactions with COVID-19 patients, PPE use was reported by the majority of participants at both assessments, with approximately 93% indicating PPE use overall (Table 5). Low rates of adherence to single-glove use were reported in both time frames. Similarly, adherence to the removal of PPE according to protocol, hand hygiene before and after patient contact, and frequent decontamination of surfaces was low, especially during the second assessment, while adherence after exposure to bodily fluids remained very high (>91%) in both assessments (Table 4). Some practices demonstrated reduced adherence in the second assessment. The proportion of HCWs reporting always removing and replacing PPE according to protocol decreased markedly from 55.2% at the first assessment to 19.7% at the second (*p* < 0.001, Table 4). Additionally, daily decontamination of frequently touched personal items showed reduced adherence over time, with the rate of HCWs who ‘always’ decontaminated decreasing from 44.3% to 27.6%.

#### 3.2.2. Adherence to IPC Measures When Performing Aerosol-Generating Procedures

During aerosol-generating procedures, adherence to several IPC measures remained high; however, key declines were observed in specific practices (Table 6). While a consistent use of the core PPE items (medical masks, gloves, and face shields or goggles) remained high overall, and hand hygiene after exposure to bodily fluids continued to exceed 90%, notable worsening was concentrated in PPE replacement and hand hygiene before patient contact. In particular, adherence to removal and replacement of PPE following aerosol-generating procedures, when considering only the “always” response, declined markedly from 58.38% at the first assessment to 50.86% at the second (*p* = 0.042). Similarly, always performing hand hygiene before and after touching a COVID-19 patient during AGPs decreased from 55.49% to 48.55% (*p* = 0.031). In addition, daily decontamination of frequently touched surfaces showed a decline, with the rate of ‘always’ decontaminating decreasing from 38.72% to 26.01% (*p* < 0.001). These findings indicate that, despite generally high PPE use, adherence to several critical IPC behaviors deteriorated over time, particularly those related to PPE replacement and pre-contact hygiene during aerosol-generating procedures.

## 4. Discussion

In the present prospective study of HCWs from public hospitals in Greece, we observed persistently high levels of occupational exposure to COVID-19, with a notable increase in the proportion of HCWs classified as high-risk over the six-month follow-up period. Despite stable rates of reported occupational exposure across assessments, the shift toward higher-risk classification may suggest a deterioration in protective conditions or practices over time rather than increased exposure frequency alone. Nurses consistently exhibited a higher exposure risk compared with physicians, reinforcing previous evidence that the professional role and the intensity of patient contact are key determinants of occupational vulnerability [5]. While female HCWs appeared to have higher exposure rates, this finding may be attributed to the predominance of women in nursing roles rather than sex-specific risk. Age was associated with occupational exposure at baseline but not at follow-up, indicating that the demographic risk patterns may be context-dependent and that they evolve over time. Importantly, although the overall use of personal protective equipment remained high, adherence to several critical IPC practices declined at follow-up, underscoring challenges in sustaining optimal protective behaviors as the pandemic progressed.

To our knowledge, prospective analyses specifically assessing occupational exposure risk among HCWs using standardized WHO-based risk assessment tools are scarce. The majority of studies evaluating occupational exposure and IPC practices during the COVID-19 pandemic have employed cross-sectional designs, which capture exposure at a single time point and do not allow assessment of temporal changes in risk classification or behavioral adherence [11,12]. In Greece, available evidence is similarly scarce. Maltezou et al. [14] examined SARS-CoV-2 infection among HCWs with high-risk occupational exposure in the context of a seven-day exclusion-from-work policy. However, that study focused on infection outcomes and occupational health policy evaluation rather than on prospective changes in exposure risk or IPC practice adherence. In contrast, our study provides prospective insight into how occupational risk classification and preventive practices of HCWs in contact with COVID-19 cases evolved over a six-month period, thereby addressing an important gap in the existing literature and contributing evidence on the sustainability of protective behaviors beyond the acute phase of the pandemic.

The increase in the proportion of HCWs classified as high-risk for COVID-19 infection at follow-up, despite stable occupational exposure rates, as estimated by the questionnaire, may suggest a shift in the quality of exposure rather than in its frequency. This finding may reflect changes in adherence to IPC measures over time, potentially driven by fatigue due to the pandemic, reduced risk perception, and normalization of COVID-19 in clinical settings. Previous studies have documented worsening adherence to protective behaviors relating to respiratory infections, particularly when perceived threat diminished or workloads increased [9,15]. Longitudinal evidence from other healthcare settings has similarly indicated that sustained behavioral adherence is difficult to maintain during prolonged public health emergencies, even when knowledge and availability of personal protective equipment remain adequate [16,17]. Additionally, increased clinical needs and staff shortages during later pandemic phases may have contributed to reduced protocol adherence, thereby elevating exposure risk. Although our study was not designed to address the underlying cause(s) of changes in risk severity for COVID-19 exposure risk, the aforementioned concepts underscore that high-risk occupational exposure may be influenced by temporal, behavioral, and organizational factors. Taken together, these findings underscore the importance of continuous monitoring and reinforcement of protective practices beyond the acute phases of a pandemic.

Despite its strength, our study has some limitations. The study population was derived using a convenience sampling approach from four public hospitals in a single geographic region, which may limit the generalizability of the results to other healthcare settings, private institutions, or regions with different organizational structures and resource availability. Although the study employed a longitudinal design, the data were collected at only two time points over a six-month interval, which may not fully capture shorter-term fluctuations in occupational exposure risk or adherence to IPC practices. The exposure risk and adherence to IPC measures were assessed using self-reported questionnaires, which are subject to recall bias and social desirability bias. However, we used the “Risk Assessment and Management of Exposure of Health Care Workers in the Context of COVID-19” tool, a standardized instrument that has been extensively used and shown to be reliable in epidemiological and occupational health studies [10]. Our study did not account for several potential confounders, including workload intensity, staffing shortages, changes in the institutional IPC policies or availability of PPE, all of which may have influenced the exposure risk and adherence patterns. Additionally, while professional category and demographic variables were examined, the sample size within some subgroups was limited, reducing statistical power for subgroup analyses. The analyses were conducted using complete-case (listwise deletion) procedures, which are standard in SPSS. Although this approach may have resulted in a slight reduction in sample size and the potential for limited sample bias if data were not missing completely at random, it was unlikely to meaningfully influence the overall conclusions of the study. We reported higher occupational risk in nurses versus physicians. The present study did not account for potential confounders such as the intensity of clinical exposure, staffing levels, or the specific healthcare unit environment, and thus we cannot clarify whether professional category was an independent risk factor.

## 5. Conclusions

In conclusion, in this prospective study of a sample of HCWs, occupational exposure to COVID-19 remained substantial, with an increase in high-risk classification over the six-month follow-up. This trend, occurring despite stable estimates for occupational exposure, suggests that occupational risk may be dynamic and may be influenced by changes in protective behaviors over time. Our findings emphasize the importance of continued surveillance, targeted interventions, and reinforcement of protective measures to mitigate occupational risk in future infectious disease threats.

## Figures and Tables

**Table 1:** Demographic and occupational characteristics of the study population.

Variables	N (%)
Age	
<35 years	20 (9.9%)
≥35 years	161 (79.3%)
Not reported	22 (10.8%)
Gender	
Male	47 (23.2%)
Female	141 (69.5%)
Not reported	5 (2.5%)
**Type of healthcare professional**	
Physician	55 (27.1%)
Registered nurse	81 (39.9%)
Nurse assistant	21 (10.3%)
Radiology technician	4 (2.0%)
Respiratory physiotherapist	4 (2.0%)
Dietician	1 (0.5%)
Midwife	5 (2.5%)
Other	31 (15.3%)

**Table 2:** Demographic parameters’ association with the exposure risk at baseline vs. at follow-up. Abbreviations: HCW, healthcare worker. The *p*-values refer to within-person changes over time.

Variable	High-Risk at Baseline (n/N, %) [95% CI]	High-Risk at Follow-Up (n/N, %) [95% CI]	*p*-Value
**Age**			
<35 years	13/20 (65%) [0.43–0.83]	14/20 (70%) [0.48–0.86]	0.777
≥35 years	89/161 (55.3%) [0.47–0.63]	98/161 (60.9%) [0.53–0.68]	0.428
**Gender**			
Male	28/47 (59.6%) [0.45–0.73]	29/47 (61.7%) [0.47–0.74]	0.166
Female	78/141 (55.3%) [0.47–0.63]	91/141 (64.5%) [0.56–0.72]	0.604
**HCW type**			
Nurses	68/81 (84%) [0.74–0.90]	65/81 (80.2%) [0.70–0.88]	<0.001
Physicians	46/55 (83.6%) [0.72–0.91]	48/55 (87.3%) [0.76–0.94]	<0.001

**Table 3:** Transitions of the occupational risk category change in the group of participants with occupational exposure (n = 148).

	Follow-Up: Low-Risk	Follow-Up: High-Risk	Total (Baseline)
Baseline: Low-risk	8 (Stable low)	27 (Increase)	35
Baseline: High-risk	10 (Decrease)	103 (Stable high)	113
Total (Follow-up)	18	130	148

**Table 4:** Occupational risk category changes by healthcare professional type. Data are expressed as absolute numbers.

Healthcare Professional Type	High-Risk to Low-Risk	Low-Risk to High-Risk
Physicians	5	6
Registered Nurses	9	10
Nurse Assistants	2	4
Dieticians	0	1
Technical Staff	0	2
Others	1	4

**Table 5:** Response rates of questions regarding IPC measure adherence during healthcare interactions.

Question	1st Assessment	2nd Assessment	*p* Value
During the period of a healthcare interaction with a COVID-19 patient, did you wear PPE? Yes/No	189 (93.10%)/14 (6.9%)	189 (93.10%)/14 (6.9%)	1.000
If yes, for each item of PPE below, indicate how often you used it:			
Single glove			
Always, as recommended	48/189 (25.39%)	57/189 30.16%)	0.281
Most of the time (≥50% but not 100%)	5/189 (2.64%)	33/189 (17.46%)	
Occasionally (20% to 50%)	8/189 (4.23%)	58/189 (30.69%)	
Rarely (less than 20%)	127/189 (67.19%)	41/189 (21.69%)	
Medical mask			
Always, as recommended	185/189 (97.88%)	187/189 (98.94%)	0.625
Most of the time (≥50% but not 100%)	2/189 (1.05%)	2/189 (1.05%)	
Occasionally (20% to 50%)	2/189 (1.05%)	0/189 (0%)	
Rarely (less than 20%)	0/189 (0%)	0/189 (0%)	
Face shield or goggles/protective glasses			
Always, as recommended	179/189 (94.70%)	187/189 (98.94%)	0.021
Most of the time (≥50% but not 100%)	9/189 (4.76%)	2/189 (1.05%)	
Occasionally (20% to 50%)	0/189 (0%)	0/189 (0%)	
Rarely (less than 20%)	1/189 (0.53%)	0/189 (0%)	
During the period of healthcare interaction with the COVID-19 patient, did you remove and replace your PPE according to protocol (e.g., when medical mask became wet, disposed of wet PPE in the waste bin, performed hand hygiene, etc.)			
Always, as recommended	112/203 (55.17%)	40/203 (19.70%)	<0.001
Most of the time (≥50% but not 100%)	49/203 (24.14%)	12/203 (5.91%)	
Occasionally (20% to 50%)	12/203 (5.91%)	54/203 (26.60%)	
Rarely (less than 20%)	16/203 (7.88%)	95/203 (46.79%)	
During the period of healthcare interaction with the COVID-19 patient, did you perform hand hygiene before and after touching theCOVID-199 patient? NR irrespective of wearing gloves.			
Always, as recommended	116/203 (57.14%)	53/203 (26.10%)	<0.001
Most of the time (≥50% but not 100%)	44/203 (21.67%)	42/203 (20.69%)	
Occasionally (20% to 50%)	23/203 (11.33%)	29/203 (14.28%)	
Rarely (less than 20%)	6/203 (2.95%)	67/203 (33.00%)	
During the period of healthcare interaction with the COVID-19 patient, did you perform hand hygiene before and after any clean or aseptic procedure was performed (e.g., inserting peripheric vascular catheters, urinary catheters, intubations, etc.)?			
Always, as recommended	119/203 (58.62%)	119/203 (58.62%)	1.000
Most of the time (≥50% but not 100%)	68/203 (33.50%)	68/203 (33.50%)	
Occasionally (20% to 50%)	9/203 (4.43%)	9/203 (4.43%)	
Rarely (less than 20%)	1/203 (0.49%)	1/203 (0.49%)	
During the period of healthcare interaction with the COVID-19 patient, did you perform hand hygiene after exposure to bodily fluids?			
Always, as recommended	186/203 (91.62%)	187/203 (92.11%)	1.000
Most of the time (≥50% but not 100%)	3/203 (1.47%)	4/203 (1.97%)	
Occasionally (20% to 50%)	2/203 (0.98%)	0/203 (0%)	
Rarely (less than 20%)	0/203 (0%)	0/203 (0%)	
During the period of healthcare interaction with the COVID-19 patient, were high-touch surfaces decontaminated frequently (at least three times daily)?			
Always, as recommended	90/203 (44.33%)	56/203 (27.58%)	<0.001
Most of the time (≥50% but not 100%)	36/203 (17.73%)	65/203 (32.02%)	
Occasionally (20% to 50%)	21/203 (10.34%)	20/203 (9.85%)	
Rarely (less than 20%)	8/203 (3.94%)	14/203 (6.89%)	

**Table 6:** Response rate of questions regarding IPC measure adherence during aerosol-generating procedures. Abbreviations: PPE, personal protective equipment.

Question	1st Assessment	2nd Assessment	*p* Value
During aerosol-producing procedures on a COVID-19 patient, did you wear PPE? Yes/No	173 (85.22%)/30 (14.77%)	173 (85.22%)/30 (14.77%)	1.000
If yes, for each item of PPE below, indicate how often you used it:			
Single glove			
Always, as recommended	43/173 (24.85%)	39/173 (22.54%)	0.548
Most of the time (≥50% but not 100%)	7/173 (4.04%)	10/173 (5.78%)	
Occasionally (20% to 50%)	3/173 (1.73%)	33/173 (19.07%)	
Rarely (less than 20%)	120/173 (69.36%)	91/173 (52.60%)	
N95 mask (or equivalent respirator)			
Always, as recommended	167/173 (96.53%)	167/173 (96.53%)	1.000
Most of the time (≥50% but not 100%)	6/173 (3.47%)	6/173 (3.47%)	
Occasionally (20% to 50%)	0/173 (0%)	0/173 (0%)	
Rarely (less than 20%)	0/173 (0%)	0/173 (0%)	
Face shield or goggles/protective glasses			
Always, as recommended	167/173 (96.53%)	167/173 (96.53%)	1.000
Most of the time (≥50% but not 100%)	5/173 (2.89%)	5/173 (2.89%)	
Occasionally (20% to 50%)	1/173 (0.57%)	1/173 (0.57%)	
Rarely (less than 20%)	0/173 (0%)	0/173 (0%)	
Disposable gown			
Always, as recommended	161/173 (93.06%)	161/173 (93.06%)	1.000
Most of the time (≥50% but not 100%)	9/173 (5.20%)	9/173 (5.20%)	
Occasionally (20% to 50%)	3/173 (1.73%)	3/173 (1.73%)	
Rarely (less than 20%)	0/173 (0%)	0/173 (0%)	
Waterproof apron			
Always, as recommended	48/173 (27.75%)	37/173 (21.38%)	0.082
Most of the time (≥50% but not 100%)	10/173 (5.780%)	12/173 (6.94%)	
Occasionally (20% to 50%)	16/173 (9.24%)	14/173 (8.09%)	
Rarely (less than 20%)	99/173 (57.22%)	110/173 (63.58%)	
During aerosol-generating procedures on a COVID-19 patient, did you remove and replace your PPE according to protocol?			
Always, as recommended	101/173 (58.38%)	88/173 (50.86%)	0.042
Most of the time (≥50% but not 100%)	38/173 (21.96%)	17/173 (9.82%)	
Occasionally (20% to 50%)	18/173 (10.40%)	23/173 (13.29%)	
Rarely (less than 20%)	13/173 (7.51%)	42/173 (24.28%)	
During aerosol-generating procedures on a COVID-19 patient, did you perform hand hygiene before and after touching theCOVID-199 patient? NR irrespective of wearing gloves			
Always, as recommended	96/173 (55.49%)	84/173 (48.55%)	0.031
Most of the time (≥50% but not 100%)	45/173 (26.01%)	30/173 (17.34%)	
Occasionally (20% to 50%)	22/173 (12.71%)	25/173 (14.45%)	
Rarely (less than 20%)	0/173 (0%)	26/173 (13.29%)	
During aerosol-generating procedures on a COVID-19 patient, did you perform hand hygiene before and after touching theCOVID-199 patient’s surroundings?			
Always, as recommended	89/173 (51.44%)	74/173 (42.77%)	0.018
Most of the time (≥50% but not 100%)	42/173 (24.27%)	33/173 (19.08%)	
Occasionally (20% to 50%)	28/173 (16.18%)	18/173 (10.40%)	
Rarely (less than 20%)	9/173 (5.20%)	43/173 (24.86%)	
During aerosol-generating procedures on a COVID-19 patient, were high-touch surfaces decontaminated frequently (at least three times daily)?			
Always, as recommended	67/173 (38.72%)	45/173 (26.01%)	<0.001
Most of the time (≥50% but not 100%)	33/173 (19.07%)	36/173 (20.80%)	
Occasionally (20% to 50%)	30/173 (17.34%)	25/173 (14.45%)	
Rarely (less than 20%)	8/173 (4.62%)	32/173 (18.50%)	

## Data Availability

The data presented in this study are not publicly available due to ethical and privacy considerations involving healthcare workers. Anonymized data may be made available from the corresponding author upon reasonable request and subject to approval by the relevant institutional and ethical committees.

## Abbreviations

The following abbreviations were used in this manuscript:

HCWs	Healthcare workers
IPC	Infection prevention and control
